# Towards a Better Understanding of MASLD: Patient Health Literacy, Illness Perception, and Awareness

**DOI:** 10.3390/diseases14040147

**Published:** 2026-04-17

**Authors:** Irini Gergianaki, Foteini Anastasiou, Sophia Papadakis, Marilena Anastasaki, Manolis Linardakis, Juan Mendive, Leen J. M. Heyens, Ger Koek, Jean Muris, Christos Lionis

**Affiliations:** 1 Clinic of Social and Family Medicine, School of Medicine, University of Crete, 70013 Heraklion, Crete, Greeceanastasakimarilena@yahoo.gr (M.A.); linman@med.uoc.gr (M.L.); 2 4th Local Primary Health Care Team, 71303 Heraklion, Crete, Greece; 3 European Society for Primary Care Gastroenterology, London E1 6HU, UK; 4 La Mina Primary Health Care Academic Centre, University of Barcelona, 08007 Barcelona, Spain; 5 IDIAP Jordi Gol Research Institute, 08007 Barcelona, Spain; 6 Faculty of Health and Life Sciences, Hasselt University, 3500 Hasselt, Belgium; 7 School of Nutrition and Translational Research in Metabolism (NUTRIM), Maastricht University, 6211 LK Maastricht, The Netherlands; 8 LCRC (-MHU), Department Future Health and Gastro-enterology and Hepatology, Ziekenhuis Oost-Limburg, 3600 Genk, Belgium; 9 Department of Family Medicine, CAPHRI Research Institute, Maastricht University, 6229 HA Maastricht, The Netherlands; 10 Department of Psychology, School of Social Sciences and Humanities, University of Limassol, Limassol 3025, Cyprus

**Keywords:** MASLD, health literacy, primary care, general practice, Europe, Greece, Spain, The Netherlands

## Abstract

Objectives: The objective of this study was to investigate metabolic dysfunction-associated steatotic liver disease (MASLD)-related awareness, health literacy (HL), and illness perception among patients at risk of MASLD in European primary care settings. Methods: Participants aged ≥50 years with either obesity, metabolic syndrome (MetS), or type 2 diabetes mellitus (T2DM), and attending general practices (GPs) in Greece, Spain, or The Netherlands were included in the study. The participants completed surveys to collect data on their socio-demographic characteristics and health habits, including the European Health Literacy Survey (HLS-E-Q16), the Brief Illness Perception Questionnaire [B-IPQ], and the Public Awareness of NAFLD Questionnaire. Results: Overall, 234 patients participated in the study (mean age: 66.5 ± 9.5 years; 45.7% were male). Among the participants, 64.5%, 66.2%, and 59.8% had a diagnosis of diabetes, obesity, and MetS, respectively. Almost one-third (27.9%) had never heard about MASLD or discussed MASLD with their GP. Twelve percent (12.1%) had never heard about cirrhosis, and 20.5% were unaware that liver disorders may cause serious health problems. Overall, 43.6% of the patients had a sufficient level of HL (score >13) with a mean score of 11.5 ± 3.3. Illness perception (B-IPQ score) was low at 41.6 ± 11.6. Significantly higher B-IPQ scores were documented for female compared to male respondents (43.1 vs. 39.8; *p* < 0.01). Multivariate analysis found that knowledge about MASLD was associated with higher HLS-E-Q16 (*p* = 0.017) and B-IPQ (*p* = 0.028) scores. Conclusions: Despite being at risk, a significant proportion of the study participants were unaware of MASLD, its risk factors, and their personal susceptibility. This study underscores the importance of enhancing patient HL and promoting prevention and risk reduction, particularly among high-risk patient populations.

## 1. Introduction

MASLD (metabolic dysfunction-associated steatotic liver disease), formerly known as NAFLD (non-alcoholic fatty liver disease), is associated with disturbed metabolic function and an increased incidence of cardiovascular diseases (CVDs), dyslipidaemia, insulin resistance, type 2 diabetes mellitus (T2DM), and hypertension, which are the components of metabolic syndrome [1,2,3,4,5,6,7]. There is increasing recognition that general practitioners (GPs) and primary care providers (PCPs) play a crucial role in the prevention, early detection (case finding), and long-term management of the MASLD spectrum [1,2,8,9,10,11]. Despite the existence of clinical guidelines, MASLD receives insufficient attention among PCPs in Europe and internationally, with a large proportion of cases remaining undiagnosed or receiving a delayed diagnosis [1,2,4,12,13,14,15].

The prevention and management of MASLD emphasise lifestyle changes and metabolic risk factor management, which both require active patient participation in meeting treatment targets (e.g., weight loss and dietary changes) [1,2]. Patient health literacy (HL), illness perception (IP), and awareness have been identified as important factors in liver disease treatment and in promoting behavioural changes and may have significant implications for doctor–patient interactions and the appropriate communication of the patient’s risk [16]. HL is defined as “people’s knowledge, motivation and competencies to access, understand, appraise, and apply health information in order to make judgments and take decisions in everyday life concerning health care, disease prevention, and health promotion” [17]. People with low HL have an increased risk of experiencing poor health outcomes [18,19,20,21] and tend to receive fewer benefits from health care services, which are often limited to acute care rather than prevention and health promotion [22,23]. Large-scale studies have identified addressing limited HL as an important challenge for health practice and policy in Europe [22]. In addition, risk and illness perception, or an individual’s perceived susceptibility to a potential health threat, has been shown to mediate health and safety-related behaviours and decisions [23,24].

There are a limited number of studies that examined patient HL, IP, and awareness related to MASLD in primary health care (PHC) settings [25,26,27,28]. Documenting the patient perspective can be important in enhancing our understanding of how to best tailor patient education and support engagement in behaviour changes. As such, the aim of this study was to explore the awareness, health literacy, and illness perception of MASLD among at-risk patients in European primary care settings. We also sought to explore how communication pathways affect risk perception among high-risk patients. This study is part of a European collaborative study to support the development of new knowledge and tools to support PC providers in the early detection and management of MASLD [https://www.nash.med.uoc.gr/ accessed on 12 December 2025]. Its main aim was to serve as formative research to inform the design of a training intervention for PCPs in Europe.

## 2. Materials and Methods

### 2.1. Study Design

A cross-sectional descriptive study was conducted among patients recruited from three European PC settings in Crete (Greece), Barcelona (Spain), and Maastricht (The Netherlands).

### 2.2. Setting

A total of 12 PC practices served as data collection sites. In each country, four general practitioners’ (GPs’) practices were selected as study settings for patient recruitment. The GPs were purposively selected to represent a range in gender, age, years of experience, and area of practice (urban/semi-urban/rural) using the following criteria:

(1) Licenced general practitioner serving in the public or private sector;
(2) PC practice is in a well-defined health area;
(3) Available list of patients registered with their practice;
(4) See a minimum of 15 patients per day in practice.

### 2.3. Participants and Eligibility Criteria

The study participants were patients at high risk for MASLD that visited the selected GPs. The patient inclusion criteria were as follows:

(1) Registered with a selected GP;
(2) Aged 50 years or older;
(3) Diagnosed with obesity, MetS, or T2DM.

The inclusion criteria were assessed by the selected GPs. Obesity was defined as a BMI of 30 kg/m^2^ or greater and was identified using physical examination. MetS and T2DM were identified from patient electronic and/or paper-based medical records, namely, whether a confirmed diagnosis of either was recorded or whether the patients are being treated for either condition. The exclusion criteria included unwillingness or inability to provide signed informed consent and complete study procedures due to cognitive impairment, dementia, and/or terminal illness.

### 2.4. Study Outcomes

Supplementary Table S1 presents the study outcomes and variables, along with the methods used to assess and categorise them during the data collection and analysis. Socio-demographic characteristics, behavioural risk factors, and biomedical indices were assessed. MASLD awareness was assessed using the Public Awareness of NAFLD questionnaire [27]. HL was assessed using the European Health Literacy Survey Questionnaire (HLS-E-Q16), with the scores categorised into three levels of HL: inadequate HL (0–8), problematic HL (9–12), and sufficient HL (13–16) [21,29]. The HLS-E-Q16 has been validated for use in Greek, Dutch, and Spanish. The total HLS-E-Q16 scores were calculated as well as separate scores for its three sub-scales (health care, disease prevention, and health promotion). The Brief Illness Perception Questionnaire (B-IPQ) was used to assess illness perception with cut-offs of <42 indicating low, 42–49 indicating moderate, and ≥50 indicating high perception of risk [30]. A validated translation of the B-IPQ was utilised, and both the total scores and scores for each of the eight sub-scales (timeline, illness concern, consequences, emotional representation, identity, personal control, treatment control, and coherence) were calculated [31,32,33]. All necessary written permissions were obtained from the developers of the questionnaires for use in the present study. The study survey used the term NAFLD, which is now referred to as MASLD.

### 2.5. Data Collection Procedures

Consecutive patients visiting the participating GPs’ practices during a 6-week recruitment period were assessed for eligibility via medical chart review. The GPs assessed patient eligibility and invited patients to participate in the study. The GP kept track of the flow of patients in the study by completing a weekly logbook that recorded the number of patients screened, found eligible, agreed to participate, and completed the study procedure. Upon providing signed informed consent, the participants were given the study survey. For patients unable to complete the survey independently (inadequate reading skills or poor eyesight), a member of the primary care team assisted them with survey completion by reading the survey questions to them and recording the responses. Behavioural risk factors and biomedical indices were determined and recorded on the case report form by the GP. The study coordinator conducted a site visit at each clinic and monitored adherence to the study protocol.

### 2.6. Data Analysis

The data were analysed using SPSS v.25 software (IBM Corp., Released 2017, IBM SPSS Statistics for Windows, Armonk, NY, USA). Absolute and relative frequency distributions of the descriptive and clinical characteristics of the participants were estimated. The normality of continuous variables (scales’ scores) was established using Blom’s method (Q-Q plot). Student’s *t*-tests and χ^2^ tests were used to compare clinical characteristics and lifestyle habits between genders. χ^2^ tests were used to assess the frequency distribution of the Awareness Scale scores based on the participants’ country of origin. Descriptive measures of the levels of knowledge (Awareness Scale), illness perception, and health literacy of the participants are presented; their reliability coefficients were assessed using Cronbach’s α, and comparisons between countries were performed using the Kruskal–Wallis test. Finally, in order to assess interaction effects with health literacy, multiple linear regression (based on the Process method (v.3.5.3) and using illness perception as a mediator factor) was used to calculate the (unstandardised) β coefficients for knowledge (about conditions that cause fatty liver) in relation to the health literacy, illness perception, and characteristics of the participants [34]. The significance level was set at 0.05.

## 3. Results

### 3.1. Demographic Characteristics

Two hundred and thirty-four (n = 234) eligible patients participated in the study (mean age: 66.5 ± 9.5 years; 54.3% were female). Table 1 summarises the participant characteristics. Almost sixty percent (59.8%) were of Greek origin (n = 140), 33.8% were Spanish (n = 79), and 6.4% (n = 15) were from The Netherlands. Recruitment in The Netherlands and Spain was significantly impacted by COVID-19-related clinic disruptions. Most participants (88.5%) lived in urban areas, and 32.5% had completed high school, while 10.3% had pursued higher education.

### 3.2. Clinical and Lifestyle Characteristics

The clinical characteristics and lifestyle factors of the study participants, by gender and overall, are presented in Table 2. Overall, 67.5% of the participants were diagnosed with obesity (defined as BMI ≥ 30, up to 84% if determined based on waist circumference). Almost sixty percent of the patients (59.8%) had been diagnosed with metabolic syndrome (MetS). Twenty-one percent (20.9%, n = 49) reported being a current smoker, and most (71.4%) reported no alcohol consumption. Significant differences were found between male and female patients in terms of both alcohol and smoking behaviours. Among those who reported alcohol use, the mean number of standard alcohol drink units per week was 13 glasses/week.

### 3.3. MASLD Awareness, Health Literacy, and Illness Perception

In total, 220 out of the 234 participants had complete data for MASLD awareness, HL, and IP. Almost one-third (27.9%) of the participants had never heard about MASLD (or NAFLD) from their personal physician. A smaller percentage of 12.1% had never heard about cirrhosis, and 20.5% were unaware that liver disorders may cause serious health problems. More than half of the participants (55.2%) lacked knowledge on the availability of treatments for MASLD.

Knowledge about the conditions that cause fatty liver was limited, with 29% of the participants unaware of any or only knew one causative factor for MASLD. The patients identified the following as the most common factors involved in the development of fatty liver disease: obesity (78.8%), high cholesterol (48.7%), and excess alcohol intake (47.4%). The mean score on the Awareness Scale was 2.5 ± 1.5, where a score of 8 indicates greater knowledge (Table 3). The mean B-IPQ score was 41.6 ±11.6. The timeline component of the B-IPQ had higher mean scores compared to the treatment control and coherence components (7.8 vs. 2.6, p < 0.001). The mean HLS-E-Q16 score was 11.5 (±3.3), with the health care component showing a higher mean score compared to health promotion (5.5 vs. 2.8, *p* < 0.001). The gender analysis found significantly higher total B-IPQ and illness concern, consequences, and emotional representation sub-scale scores for females versus males. No other gender differences were found.

**Figure 1:**
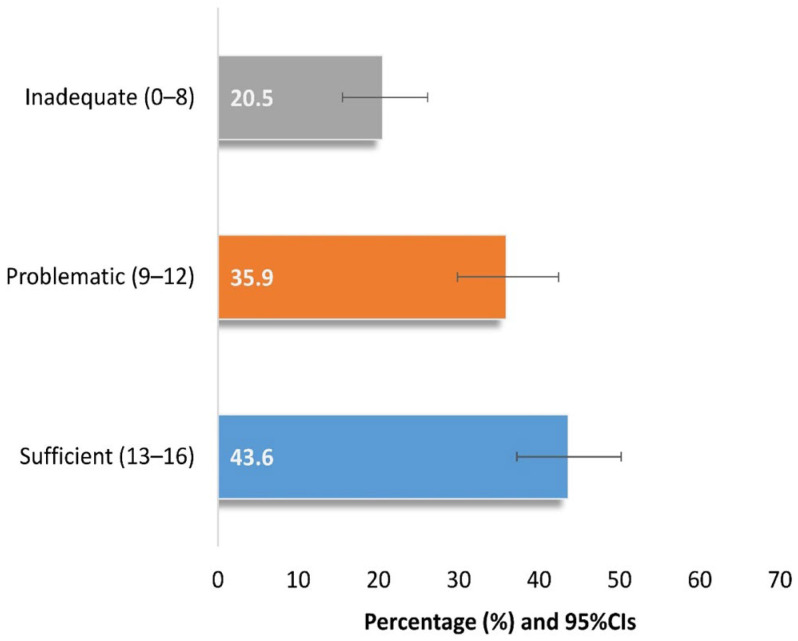
Health literacy distribution of study participants (n = 220).

In total, only 43.6% of the participants had a sufficient level of HL (score of 13–16), as depicted in Figure 1.

### 3.4. Factors Affecting MASLD Awareness

**Figure 2:**
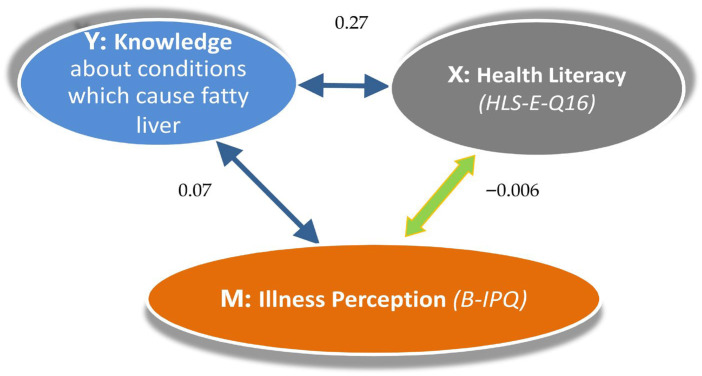
Non-standardised β coefficients (effects) of basic prognostic factors for knowledge about the conditions that cause fatty liver.

The multiple linear regression analysis of knowledge (about the conditions that cause fatty liver) in relation to the participants’ HL, IP, and characteristics is presented in Table 4. Knowledge about MASLD causative factors was significantly associated with HL (β = 0.27, *p* = 0.017), IP (β = 0.07, *p* = 0.028), their negative interaction effect (β = −0.006, *p* = 0.016), and younger age (β = −0.023, *p* = 0.033). Knowledge was lower in the participants from Greece compared to the other countries (β = −1.44, *p* < 0.001). Figure 2 presents the coefficients (effects) for HL and knowledge about the conditions that increase the probability of fatty liver, mediated by IP.

## 4. Discussion

### 4.1. Main Findings and Comparison with the International Literature

Overall, this cross-sectional survey documented low to moderate levels of MASLD-related awareness, HL, and IP in the at-risk MASLD primary care patient populations in this study. Almost one-third of patients had never heard about MASLD from their personal physician, and 20.5% reported being unaware that liver disorders may cause health issues. Validated tools for estimating awareness, IP, and HL for MASLD found low to moderate levels in this study sample. In total, 43.6% of patients had a sufficient level of HL. Significantly greater illness perception was documented among female respondents than among males, particularly for the illness concern, consequences, and emotional representation sub-scales. Given the role of patient engagement in prevention and early management through lifestyle changes and risk factor modification, these results support strengthening patient awareness and HL [1,2,35]. While there have been studies assessing PCP MASLD-related awareness [9,13,36,37,38,39,40,41], population-based studies in primary care settings that estimate awareness of MASLD are relatively limited [25,26,27,28]. A recent study in the US, which included 11,700 adults, found that, from 2007–2008 to 2015–2016, although the awareness of liver disease among adults already diagnosed with MASLD improved from 4.4% to 6.3%, it was still 4 to 10 times lower than the awareness of viral hepatitis [28]. Further studies in interventional settings, primarily outside of Europe, show low risk perception regarding MASLD in the community [25,26,27]. Additionally, there are studies that assessed HL among patients with liver disease but there is only one on patients with MASLD [42,43,44,45,46,47,48,49]. A recent study by Saddic et al. reported on HL in a sample of 101 patients attending an ambulatory hepatology clinic [49]. The study found comparable HL scores to those of the present study. The authors observed an association between lower HL and education and more advanced MASLD and metabolic dysfunction.

### 4.2. Strengths and Limitations

To the best of our knowledge, this is the first cross-sectional study to report on MASLD awareness, HL, and IP among high-risk patients in primary care practice settings. However, the study findings should be interpreted in light of the study’s limitations. Firstly, the study sample was ultimately smaller than the original sample (n = 300) due to COVID-19-related clinic disruptions during the data collection period and the resulting delays in data collection in The Netherlands. As such, the overall results and cross-country comparisons should be interpreted with caution. The present study served as formative research to inform the design of new training programmes for PCPs and subsequent interventions in PHC settings.

The study timeline involved screening patients until a target of 100 patients per county was reached; a maximum of 6 weeks of data collection per PCP was allowed before recruitment was discontinued. Further limitations include the consecutive rather than random selection of study participants, the self-reported nature of the questionnaires, and the descriptive, cross-sectional study design, which does not allow for any causal examination. Furthermore, this study represents a cross-sectional sample in a defined group of PHP practices; therefore, the study findings may not be generalisable beyond this study population.

### 4.3. Study Implications

Internationally, there has been a call for efforts to increase both PCP and patient HL related to fatty liver disease, with an emphasis on patients at high risk [1,2,4,8,50]. Research suggests that health interventions that successfully incorporate risk perceptions can lead to better behavioural change outcomes [28]. Incorporating these concepts into clinical practice, particularly in PHC, could increase the provision of understandable and accessible information, thereby increasing the impact, uptake, and sustainability of clinical interventions. As the prevalence and burden of MASLD continues to rise in parallel with the global epidemic of obesity and type 2 diabetes mellitus, public health policies should incorporate patient education to facilitate proactiveness, prompt risk stratification, and early diagnosis. These interventions should be informed by a good understanding of current patient perspectives and through monitoring of changes in key areas such as HL and IP over time.

Both the data generated and the methodologies used in the present study provide information and tools that could support future assessments and monitoring of trends in patient HL over time and across EU countries. The study findings can also be utilised to inform European and national policies on raising MASLD awareness among high-risk patients and on the emerging role of PCPs in patient education and shared decision-making. A patient guidance document on MASLD was recently published, which provides insights and lay language to support conversations and efforts to address patient MASLD-related health literacy [50]. The findings of the present study and the importance of PCPs’ efforts in increasing HL among at-risk patients have also been reflected in recently published primary practice recommendations, new PCP training programmes, and MASLD clinical care pathways published by our team in partnership with the European Society for Primary Care Gastroenterology [51,52,53].

At the time of data collection, The Netherlands had implemented an MASLD screening and treatment pathway for primary care, and some efforts from the National Medical Association had been made to raise awareness among medical practitioners. Spain had also developed an MASLD primary care pathway, whereas Greece had introduced a quality assurance and patient-safety project that has recently impacted PHC. The study design did not allow for examination of the associations between efforts to increase PCPs’ awareness and practices related to MASLD and how these efforts may influence patient awareness and health literacy. We do expect a positive relationship between PCPs’ awareness and patient health literacy. Future research should assess both within-country and between-country differences, taking into consideration the efforts being made by PC and national health systems.

The study’s findings may inform the rational planning of PHC services at the national or regional level to reduce health inequalities. For instance, inadequate HL and low levels of awareness in Crete, Greece, may partly account for the high morbidity and mortality associated with liver cancer, as reported in a recent study [54]. Furthermore, the need to enhance both health literacy and nutritional literacy (NL), particularly among individuals with lower educational attainment and lower annual incomes, has been emphasised in previous research and should be prioritised by health care stakeholders and policymakers [55]. In this context, investment in the development of targeted training programmes for PHC practitioners is strongly recommended.

## 5. Conclusions

The study documented a lack of awareness, HL, and risk perception among patients at high risk of MASLD in European primary care settings. Future research is needed to further examine patient HL, IP, and awareness in primary care settings. Importantly, monitoring trends in these areas could increase our understanding of time trends in order to inform policy and practice.

## Figures and Tables

**Table 1:** Descriptive characteristics of study participants (n = 234).

			n	%
Country	Greece	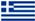	140	59.8
	The Netherlands	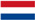	15	6.4
	Spain	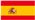	79	33.8
Gender	Males/females		107/127	45.7/54.3
Age, years	Mean ± stand. dev. (min, max)		66.5 ± 9.5 (50, 86)	
Education	Elementary school		134	57.2
	High school		76	32.5
	College, university		24	10.3
Monthly household income, Euros	Mean (median) [min, max]		1079 (1000) [0, 6650]	
Area of residence	Urban/rural		207/27	88.5/11.5

**Table 2:** Clinical characteristics and lifestyle factors of study participants by gender (n = 234).

		Total	Gender	
			Males	Females
		Mean ± SD		
Body mass index, kg/m^2^		32.9 ± 5.8	31.5 ± 5.2 *	34.0 ± 6.0
	Normal/overweight (<30.0)	76 (32.5) ^a^	46 (43.0) *	30 (23.6)
	Obese (30.0+)	158 (67.5)	61 (57.0)	97 (76.4)
Waist circumference, cm		109.5 ± 13.6	111.0 ± 13.1	108.4 ± 14.0
	Normal (<88/102)	36 (16.0)	26 (26.0) *	10 (8.0)
	Overweight/obese (88/102+)	189 (84.0)	74 (74.0)	115 (92.0)
Smoking	Current smoker	49 (20.9)	28 (26.2) *	21 (16.5)
	Former smoker	84 (35.9)	52 (48.6)	32 (25.2)
	None	101 (43.2)	27 (25.2)	74 (58.3)
Alcohol consumption ^b^	Normal	167 (71.4)	61 (57.0) *	106 (83.5)
	Increased	67 (28.6)	46 (43.0)	21 (16.5)
Number of behavioural risk factors ^c^	None	47 (20.1)	26 (24.3) *	21 (16.5)
	1	108 (46.2)	33 (30.8)	75 (59.1)
	2+	79 (33.7)	48 (44.9)	31 (24.4)

SD = standard deviation. ^a^ n (%). ^b^ Normal: <14 (for men) and <7 (for women) standard drinks per week; increased: ≥14 (for men) and ≥7 (for women) standard drinks per week. ^c^ Number of the following behavioural risk factors: obesity, smoking status, and alcohol consumption. Student’s *t*- or χ^2^ tests between genders: * *p* < 0.05.

**Table 3:** Overall Awareness Scale, illness perception, and health literacy scores and by gender (n = 220).

Scale and Component		Mean	Stand. Dev.	Median	Cronbach’s α	Males	Females	
						Mean ± SD		*p*-Value
Knowledge about conditions that cause fatty liver (Awareness Scale) ^a^		2.5	1.5	2.0	--	2.5 ± 1.4	2.5 ± 1.5	0.877
Brief Illness Perception Questionnaire (B-IPQ) ^b^	Timeline	7.8	2.4	9.0		7.7 ± 2.6	7.9 ± 2.2	0.854
	Illness Concern	6.6	2.8	7.0		6.1 ± 2.8	7.0 ± 2.7	0.007
	Consequences	6.5	2.7	7.0		6.1 ± 2.7	6.8 ± 2.7	0.033
	Emotional representation	6.3	3.0	7.0		5.8 ± 2.8	6.8 ± 3.1	0.002
	Identity	5.3	2.9	6.0		5.1 ± 2.8	5.4 ± 3.0	0.325
	Personal control	3.9	2.4	4.0		3.8 ± 2.4	3.9 ± 2.4	0.811
	Treatment control	2.6	2.1	2.0		2.6 ± 2.3	2.7 ± 2.0	0.349
	Coherence	2.6	2.3	2.0		2.6 ± 2.3	2.6 ± 2.3	0.998
	Total B-IPQ	41.6	11.6	43.0	0.730	39.8 ± 11.1	43.1 ± 11.8	0.013
European Health Literacy Questionnaire 16 (HLS-E-Q16) ^c^	Health care	5.5	1.6	6.0	0.829	5.7 ± 1.5	5.3 ± 1.6	0.093
	Disease prevention	3.3	1.3	3.0	0.743	3.4 ± 1.3	3.3 ± 1.3	0.586
	Health promotion	2.8	1.2	3.0	0.733	2.7 ± 1.2	2.8 ± 1.2	0.801
	Total HLS-E-Q16	11.5	3.3	12.0	0.902	11.8 ± 3.3	11.3 ± 3.4	0.347

SD = standard deviation. ^a^ Answer to question 4 of the Awareness Scale (Which of these conditions do you think cause fatty liver?); score is the number of correctly identified conditions out of eight, where a higher score reflects better knowledge. ^b^ Responses to the 8-component questionnaire range from 0 to 10, where a higher score reflects a more threatening perception of the illness. Friedman test between the 8 components: *p* < 0.001. ^c^ Responses to the 16-item questionnaire range from 0 to 16 (0 to 4 for each component), where a higher score reflects a sufficient level of literacy (n = 220). Friedman test between the three components: *p* < 0.001. Mann–Whitney tests used to compare genders.

**Table 4:** Multiple linear regression analysis of awareness in relation to health literacy, illness perception, and characteristics of study participants.

Model	β (95% CI)	Stand. Error	t	*p*-Value
Knowledge about conditions that cause fatty liver				
Constant a	4.31 (0.15, 8.46)	2.11	2.04	0.042
Health literacy (HLS-E-Q16)	0.27 (0.05, 0.49)	0.11	2.40	0.017
Illness perception (B-IPQ)	0.07 (0.01, 0.13)	0.03	2.22	0.028
Health literacy x illness perception	−0.006 (−0.011, −0.001)	0.002	−2.42	0.016
Gender (1: male; 2: female)	−0.09 (−0.47, 0.30)	0.19	−0.44	0.658
Age (years)	−0.023 (−0.044, −0.002)	0.01	−2.14	0.033
Education (1: elementary school; 2: high school; 3: college or university)	0.18 (−0.14, 0.50)	0.16	1.12	0.262
Behavioural risk factors (obesity, smoking, and alcohol consumption)	−0.27 (−0.55, 0.01)	0.14	−1.91	0.056
Country 1 (Greece vs. others)	−1.44 (−2.28, −0.61)	0.43	−3.40	<0.001
Country 2 (Spain vs. others)	−0.77 (−1.68, 0.12)	0.46	−1.70	0.090
	F(9, 210) = 3.30, *p* < 0.001, R^2^ = 0.124			

The linear regression analysis was based on the Process method (v.3.5.3) [35] and used illness perception as a mediator factor to assess interaction effects with health literacy.

## Data Availability

Dataset available on request from the authors.

## Supplementary Materials

The following supporting information can be downloaded at: https://www.mdpi.com/article/10.3390/diseases14040147/s1, Table S1. Study outcomes and variables assessment and categorisation.
