# Supplementary material for: Towards a Better Understanding of MASLD: Patient Health Literacy, Illness Perception, and Awareness
Source: Diseases. 2026 Apr 17;14(4):147. doi: 10.3390/diseases14040147 (PMC13115058; doi:10.3390/diseases14040147)
Supplement: Supplementary file 1 [file diseases-14-00147-s001.zip › diseases-4224236-supplementary.pdf]

Table S1. Study outcomes and variables assessment and categorisation.

**Primary Outcomes**	**Assessment**	**Categorization**
Level of health literacy	European Health Literacy Survey Questionnaire - 16 (25)	Health Literacy Survey Score: inadequate HL (0-8), problematic HL (9-12), sufficient HL (13-16)
Illness perception	Brief Illness Perception Questionnaire (31-34)	Illness Perception: Cognitive perception of illness (consequences, timeline, personal control, treatment control, identity), Emotional perception of illness (concern, emotions), Comprehension about illness, Perceived causes
MASLD awareness	Public Awareness of NAFLD Questionnaire (18)	-
Secondary outcome	Assessment	Categorization
Communication pathways affecting risk perception	Narrative literature review	-
Sociodemographic characteristics	Assessment	Categorization
Gender	Patient-reported answer in: male, female & non-binary	Gender (male, female, non-binary)
Age	Patient-reported answer in: Years (absolute value)	Age groups (50-59, 60-69, 70-79, 80+)
Education	Patient-reported answer in the highest level completed: Elementary School, High School, College/University, Postgraduate	Educational level: Low (primary, lower and middle [both prevocational secondary), Moderate (secondary vocational, senior general secondary and pre-university), High (higher professional and university Bachelor, University Master)
Monthly income	Patient-reported answer in: Euros (absolute value)	Monthly Income: Low (<1,750€), Moderate (1,750-3,000€), High (>3,000€)
Area of residence	Patient-reported answer in: Urban, Semi-urban, Rural	Residence: Urban, Semi-urban, Rural
Behavioural risk factors	Assessments	Categorization
Smoking status	Patient-reported answer in: Current-smoker, Former-smoker, Non-smoker	Smoking: Current-smoker, Former-smoker, Non-smoker
Smoking severity (for current smokers)	Patient-reported answer in: Number of cigarettes smoked per day	Smoking severity: Light (<10 cigarettes per day), Moderate (10-20 cigarettes per day), Heavy smoker (>20 cigarettes per day)
Alcohol consumption	Patient-reported answer in: Number of standard alcohol drinks consumed per week (*standard drink: a glass of water or a glass of wine or a shot)*	Alcohol consumption: Normal [<14 (for men) and <7 (for women) standard drinks per week], Increased [≥14 (for men) and ≥7 (for women) standard drinks per week]
Biomedical indexes	Assessment	Categorization
Weight	Physical examination: Kilograms (absolute value)	-
Height	Physical examination: Centimetres (absolute value)	-
Body Mass Index	Physical examination: kg/m^2 (absolute value)	BMI: Underweight (<18.5), Normal (18.5-24.9), Pre-obesity (25.0-29.9), Obese (≥30)
Waist circumference	Physical examination: Centimetres (absolute value)	Abdominal obesity: Yes (waist circumference ≥102 centimetres for males and ≥88 centimetres for females), No (waist circumference<102 centimetres for males and <88 centimetres for females)
